# Clinical effectiveness of dupilumab in CRSwNP: unaffected by baseline nasal polyp size in real-world settings

**DOI:** 10.1007/s00405-025-09275-2

**Published:** 2025-02-20

**Authors:** Michael Habenbacher, Ulrich Moser, Ahmed Abaira, Peter Kiss, Clemens Holzmeister, Jakob Pock, Katharina Walla, Angelika Lang, Alexandros Andrianakis

**Affiliations:** Department of Otorhinolaryngology, Medical University of Graz, Graz, Austria

**Keywords:** Chronic rhinosinusitis, CRSwNP, Biological treatment, Monoclonal antibody

## Abstract

**Purpose:**

This study aimed to investigate the impact of baseline nasal polyp score (NPS) on the effectiveness of dupilumab treatment in patients with chronic rhinosinusitis with nasal polyps (CRSwNP).

**Methods:**

In this retrospective observational study, 80 CRSwNP patients treated with dupilumab 300 mg biweekly at a tertiary referral center were stratified according to the baseline NPS into two groups: low-NPS (< 5) and high-NPS (≥ 5). Treatment outcomes were evaluated at the 6-month follow-up visit and compared.

**Results:**

Both groups showed significant clinical improvements. The NPS decreased significantly in both low- and high-NPS groups, from a mean score of 3.2 to 0.8 and from 6.1 to 1.4, respectively (*p* < 0.001 for both). SNOT-22 scores improved significantly in both groups (*p* < 0.001 for both), though the reduction was greater in the high-NPS group (35.5 vs. 23.9, *p* = 0.018). There were no significant differences between low- and high NPS groups in proportions of NPS reduction of ≥ 1 (89% vs. 95%, *p* = 0.396) and clinically significant SNOT-22 improvement (= reduction > 12 or follow-up SNOT < 40; 80% vs. 86%, *p* = 0.544).

**Conclusions:**

Our results suggests that dupilumab is effective in CRSwNP treatment, regardless of baseline nasal polyp size. Both small and large polyp groups showed significant improvements in NPS and patient-reported outcome measures. Future, prospective studies are warranted to validate these findings.

## Introduction

Chronic rhinosinusitis with nasal polyps (CRSwNP) is a substantial health challenge that affects approximately 2–4% of the population in Western countries [1, 2]. Recent advances in understanding the pathophysiology of CRSwNP have paved the way for biologic therapies that target specific drivers of type 2 inflammation. These therapies include monoclonal antibodies, which inhibit key cytokines and pathways involved in disease progression. Dupilumab, approved as the first biologic for CRSwNP, works by blocking the IL-4Rα receptor, thereby interrupting the signaling pathways of IL-4 and IL-13. Clinical trials and real-world studies have demonstrated the effectiveness of dupilumab in reducing nasal polyp size, improving symptoms, and enhancing patients’ overall quality of life [3, 4].

In order to identify CRSwNP patients suitable for biologics, European Guidelines on indication and evaluation of biologic treatment (= EPOS/EUFOREA criteria) were developed. The criteria for defining eligibility for biologic therapies in CRSwNP are continually being refined [5]. Currently, biologics are typically reserved for patients with severe CRSwNP, commonly defined as a total nasal polyp score (NPS) > 4 on a 0–8 scale [6]. Presumably, this threshold is primarily oriented on the eligibility criteria used in the respective randomized controlled trials [7] and similar cut-off values has been adopted as a reimbursement requirement in some countries, such as Italy and Hungary [8, 9]. However, a recent meta-analysis of 55 studies demonstrated a lack of correlation between NPS and patient-reported symptom scores, suggesting that the current endoscopic grading systems do not fully capture the subjective symptom burden [10]. In clinical practice, patients with smaller polyps often also report significant symptoms and reduced quality of life. In contrast to strict NPS-based criteria, the Austrian Federation of Social Insurances does not enforce a specific NPS threshold for dupilumab reimbursement [7]. A recent Austrian study reported that baseline NPS did not affect the efficacy of dupilumab treatment [11]. Similarly, at our Austrian tertiary referral center, dupilumab treatment is prescribed for CRSwNP patients without a strict cut-off value of nasal polyp size, allowing a more individualized approach to care.

This study aimed to evaluate the effectiveness of dupilumab treatment in CRSwNP patients with smaller polyps (NPS < 5) and larger polyps (NPS ≥ 5). We hypothesized that both groups experience a significant treatment outcome indicating that dupilumab is effective in CRSwNP treatment independent from baseline nasal polyp size.

## Materials and methods

### Study conduct and participants

This retrospective, longitudinal, observational study was carried out at the Department of Otorhinolaryngology from a tertiary referral center in Austria. A total of 80 adult patients diagnosed with primary CRSwNP, who were treated with dupilumab 300 mg biweekly between 2020 and 2024 were included in this study. Evaluation of dupilumab treatment outcome was performed at the six-month follow-up visit.

### Indication criteria for dupilumab therapy

At our institution, CRSwNP patients were prescribed dupilumab according to Austrian Federation of Social Insurances and the following modified EPOS criteria [5]: presence of nasal polyps, prior endoscopic sinus surgery (ESS) or contraindications to surgery, along with at least three of the following: (1) evidence of type 2 inflammation defined by eosinophil counts (EOS) ≥ 0.25 × 10⁹/L or total serum IgE ≥ 100 kU/L, (2) requirement for systemic corticosteroids (sCOS) in the last years, (3) baseline Sinonasal Outcome Test (SNOT-22) score ≥ 40, (4) reduced olfactory function (scored ≥ 1 on the “smelling” subitem in SNOT-22) and (5) comorbid asthma or N-ERD.

### Examination procedures

At baseline and at six-month follow-up, participants underwent a complete ENT examination including a nasal endoscopy for nasal polyp size assessment, using the Meltzer-NPS, which rates nasal polyp burden from 0 to 8 [12]. Participants were asked at each visit to fill out the German version of the SNOT-22 [6]. Furthermore, demographics and baseline characteristics included age, sex, number of prior ESS, serum EOS, serum total IgE, presence of asthma and N-ERD were also obtained.

### Study objective

Included subjects were stratified according their baseline NPS into a low-NPS group (baseline NPS of < 5) and a high-NPS group (baseline NPS of ≥ 5). The main aim of this study was to evaluate the dupilumab treatment outcome for each NPS group separately. Further, we compared treatment outcome between NPS groups. Outcome measures included: clinically relevant NPS reduction = NPS reduction of ≥ 1; clinically relevant SNOT-22 improvement = SNOT-22-reduction of > 12 [minimal clinically important difference (MCID)] or a follow-up SNOT-22 < 40, in case of a baseline SNOT-22 < 40: only the MCID applied as criterion [5, 13]; absolute NPS reduction; absolute SNOT-22 reduction, absolute reduction in SNOT-22 rhinologic subitems: nasal blockage score, impaired sense of smell score, rhinorrhea score and post-nasal drip score.

## Results

### Demographics and baseline clinical characteristics

Among the total cohort, 35 patients (44%) had a baseline NPS < 5, while 45 patients (56%) had a baseline NPS ≥ 5. Comprehensive demographics and baseline characteristics are summarized in Table 1. Except for a significant difference in sex distribution (*p* = 0.024), no other significant differences in demographics or baseline clinical characteristics were observed between the NPS groups.

**Table 1:** Demographic and baseline clinical characteristics stratified by NPS groups

Characteristic	Low-NPS group (*n* = 35)	High-NPS group (*n* = 45)	*p*-value
Sex, w/m	21/14	15/30	0.024
Age at start of treatment	51 (SD 16.2)	50.8 (SD 13.4)	0.983
n° of prior ESS	2.4 (SD 1.3)	2.4 (SD 1.7)	0.964
Duration between last ESS and dupilumab initiation	48.2 (SD 49)	43 (SD 51.8)	0.652
Evidence of type 2 inflammation	32 (91%)	44 (97%)	0.314
Serum EOS (x10⁹/L)	0.4 (SD 0.3)	0.6 (SD 1.1)	0.281
Serum total IgE (kU/L)	170.8 (SD 270)	221.4 (SD 202)	0.341
Baseline SNOT-22	50.4 (SD 16.9)	59.5 (SD 23.1)	0.054
Baseline SNOT-22 ≥ 40	25 (71%)	37 (82%)	0.251
Nasal obstruction score	3.6 (SD 1.1)	3.8 (SD 1.2)	0.561
Impaired sense of smell score	3.6 (SD 1.5)	4.2 (SD 1.3)	0.057
Rhinorrhea score	2.8 (SD 1.4)	3 (SD 1.5)	0.554
Post nasal drip score	3 (SD 1.6)	3.3 (SD 1.3)	0.381
Coexisting asthma	26 (74%)	28 (62%)	0.253
Coexisting N-ERD	5 (14%)	10 (22%)	0.367

Continuous variables are presented as means + standard deviations (SD). Categorical variables are presented as absolute numbers and percentages (%). w: women. m: men. ESS: endoscopic sinus surgery. COS: corticosteroids. N-ERD: NSAID-exacerbated respiratory disease. SNOT-22: Sinonasal outcome test 22

### Treatment outcome of patients with smaller nasal polyp size

Of the 35 patients in the low-NPS group, 31 (89%) achieved a clinically relevant reduction in NPS. Additionally, 28 patients (80%) reported a clinically relevant SNOT-22 improvement. The mean NPS decreased significantly by 2.4 points (95%CI: 1.9–2.8), from a baseline of 3.2 (SD 1.1) to 0.8 (SD 1.1) (*p* < 0.001). The mean SNOT-22 score showed a significant reduction of 23.9 points (95%CI: 17.1–30.6), decreasing from 50.4 (SD 16.9) at baseline to 26.4 (SD 19.8) (*p* < 0.001). All individual symptom scores also showed significant improvements (*p* < 0.001). Detailed results are provided in Table 2.

**Table 2:** Dupilumab treatment outcome of low-NPS group (*n* = 35)

Parameter	Baseline	6-month follow up	Mean diff. [95% CI]	*p*-value
NPS	3.2 (SD 1.1)	0.8 (SD 1.1)	2.4 [1.9–2.8]	< 0.001
SNOT-22	50.4 (SD 16.9)	26.4 (SD 19.8)	23.9 [17.1–30.6]	< 0.001
Nasal obstruction score	3.6 (SD 1.1)	1.8 (SD 1.2)	1.8 [1.3–2.2]	< 0.001
Impaired sense of smell score	3.6 (SD 1.5)	1.7 (SD 1.3)	1.9 [1.3–2.4]	< 0.001
Rhinorrhea score	2.8 (SD 1.4)	1.2 (SD 1.2)	1.5 [1.0-1.9]	< 0.001
Post nasal drip score	3 (SD 1.6)	1.6 (SD 1.3)	1.3 [0.8–1.8]	< 0.001

Continuous variables are presented as mean + standard deviation (SD) or 95% confidence interval [CI]. NPS: nasal polyp score. SNOT-22: Sinonasal outcome test 22

### Treatment outcome of patients with larger nasal polyp size

Among the 45 patients in the high-NPS group, 43 (95%) experienced a clinically significant reduction in NPS. The mean NPS decreased significantly by 4.6 points (SD 2), from a baseline of 6.1 (SD 1) to 1.4 (SD 1.6) (*p* < 0.001). Furthermore, 39 patients (86%) achieved a clinically significant SNOT-22 improvement. The mean SNOT-22 score showed a significant decline of 35.5 points (SD 22.4), dropping from 59.5 (SD 23.1) at baseline to 23.9 (SD 19.1) (*p* < 0.001). All individual symptom scores also improved significantly (*p* < 0.001). Comprehensive data are presented in Table 3.

**Table 3:** Dupilumab treatment outcome of high-NPS group (*n* = 45)

Parameter	Baseline	6-month follow up	Mean diff. [95% CI]	*p*-value
NPS	6.1 (SD 1.0)	1.4 (SD 1.6)	4.6 [4.0-5.2]	< 0.001
SNOT-22	59.5 (SD 23.1)	23.9 (SD 19.1)	35.5 [28.7–42.2]	< 0.001
Nasal obstruction score	3.8 (SD 1.2)	1.6 (SD 1.4)	2.2 [1.7–2.6]	< 0.001
Impaired sense of smell score	4.2 (SD 1.3)	1.6 (SD 1.7)	2.6 [2.0-3.1]	< 0.001
Rhinorrhea score	3 (SD 1.5)	1.1 (SD 1.2)	1.8 [1.4–2.3]	< 0.001
Post nasal drip score	3.3 (SD 1.3)	1.4 (SD 1.4)	1.8 [1.3–2.3]	< 0.001

Continuous variables are presented as mean + standard deviation (SD) or 95% confidence interval [CI]. NPS: nasal polyp score. SNOT-22: Sinonasal outcome test 22

### Treatment outcome comparison between NPS-groups

No significant differences in clinically relevant NPS reduction (89% vs. 95%, *p* = 0.396) and SNOT-22 improvement (80% vs. 86%, *p* = 0.544) were observed between NPS groups. Patients in the high-NPS group showed a significantly greater absolute NPS reduction and SNOT-22 reduction (35.5 vs. 23.9, *p* = 0.018) compared to patients with a low-NPS. There were no significant differences in all individual symptom scores between NPS groups (*p* > 0.05). Detailed results are depicted in Table 4.

**Table 4:** Dupilumab treatment outcome comparison between NPS-groups

Parameter	Low-NPS group (*n* = 35)	High-NPS group (*n* = 45)	Mean diff. [95% CI]	*p*-value
Clinically relevant NPS reduction (≥ 1)	31 (89%)	43 (95%)	6%	0.396
Clinically relevant SNOT-22 improvement*	28 (80%)	39 (86%)	6%	0.544
Absolute NPS reduction	2.4 (SD 1.3)	4.6 (SD 2)	2.2 [1.4-3]	< 0.001
SNOT-22 reduction	23.9 (SD 19.6)	35.5 (SD 22.4)	11.6 [2.1–21]	0.018
Nasal obstruction score reduction	1.8 (SD 1.3)	2.2 (SD 1.6)	0.4 [-0.3-1]	0.279
Sense of smell score reduction	1.9 (SD 1.6)	2.6 (SD 1.8)	0.7 [0.0-1.4]	0.090
Rhinorrhea score reduction	1.5 (SD 1.3)	1.8 (SD 1.5)	0.3 [-0.2-1]	0.257
Post nasal drip score reduction	1.3 (SD 1.5)	1.8 (SD 1.6)	0.5 [-0.1-1.2]	0.139

Continuous variables are presented as mean + standard deviation (SD) or 95% confidence interval [CI]. NPS: nasal polyp score. SNOT-22: Sinonasal outcome test 22. * SNOT-22 reduction > 12 or follow-up SNOT-22 < 40

## Discussion

Dupilumab was the first biologic approved for CRSwNP treatment in 2019 [3]. To assist physicians in clinical decision-making and address the high cost of biological treatment, an expert-panel subsequently established European guidelines to help identify patients most likely to benefit from biologic therapy [14]. These guidelines recommend biologics for CRSwNP patients with bilateral nasal polyps who have undergone previous sinus surgery and meet at least three of the following five criteria: evidence of type-2 inflammation, a history of systemic COS use in recent years, impaired quality of life, significant loss of smell, and comorbid asthma. Over time, these criteria have been refined, e.g. with the addition of specific cut-off values to enhance their precision [5]. Besides these international recommendations, the national health care agencies of the individual countries have introduced their own criteria for the reimbursement of biologics. Some countries enforce a certain baseline NPS level for biologic reimbursement. For instance, Italy requires an NPS of at least 5 [8], while Hungary mandates a minimum score of 4 [9]. In contrast, the Austrian Federation of Social Insurances does not enforce a strict NPS cutoff, allowing for a more individualized approach to treatment decisions [7]. In our clinical experience, patients with smaller polyps can also suffer from significant symptom burden and impaired quality of life. Therefore, we have prescribed dupilumab treatment from the outset without adhering to a strict NPS threshold, as we believe these patients can also benefit from the therapy. As expected, the greater proportion of our patients had larger polyps. However, 71% of those with smaller polyps had a SNOT-22 > 40, highlighting their considerable symptom burden. Furthermore, while patients with larger polyps showed slightly higher baseline SNOT-22 scores, the difference was neither statistically nor clinically significant. Several studies have assessed the effectiveness of dupilumab in CRSwNP in real-life settings [4, 15]. However, there is only one study that specifically assessed the outcome of dupilumab in CRSwNP patients with small nasal polyps and compared them to patients with larger nasal polyps [11]. In this Austrian study, the authors reported that size of baseline NPS did not affect the efficacy of dupilumab treatment. Similar to our Austrian institution, they prescribed dupilumab in CRSwNP independent from baseline nasal polyp size. The authors found that patients with smaller polyps (NPS ≤ 4) and those with larger polyps (NPS ≥ 5) both achieved significant improvements in NPS and SNOT-22. We observed comparable results in our study, with both low-NPS and high-NPS groups demonstrating significant reductions in NPS, SNOT-22, and individual symptom scores. Campion et al. [11] employed a linear mixed model for group comparison, demonstrating that the reduction in NPS and improvement in various quality of life measures were independent of baseline NPS. However, the authors did not evaluate and compare the response criteria: NPS reduction (≥ 1) and quality of life improvement (SNOT-22 < 40 + > MCID), as recommended by EPOS/EUFOREA [5]. Moreover, the NPS response criterion serves in Austria as national reimbursement requirement [7]. Hence, the percentage of their patients in both groups who were able to continue therapy is not reported, in contrast to our study. Our analysis revealed no significant differences between patients with smaller polyps and those with larger polyps in clinically significant NPS reduction and SNOT-22 improvement. Patients with larger polyps exhibited a greater absolute reduction in SNOT-22. However, it should be noted that higher baseline SNOT-22 values tend to drop easier and the observed statistically significant difference remains below the MCID of 12 [13]. Moreover, there were no significant differences in improvements of the individual symptom scores between the groups. On the other hand, patients with larger nasal polyps showed a significant higher absolute NPS reduction. This is not unexpected, as their larger polyp size offers a greater potential range for reduction. However, a recent meta-analysis including 55 studies revealed no correlation between current NPS gradings and patient-reported outcome [10]. Current grading systems has several limitations, including the non-linearity, the dynamic range of the quantitative scoring and the uni-dimensionality [16]. These aspects, Campion et al. [11], and our novel results support the rationale for avoiding a strict NPS threshold when prescribing dupilumab, at least until nasal polyp grading systems with improved clinical utility are developed. However, even though dupilumab may be effective independent of baseline nasal polyp size, treatment decisions should carefully weigh several factors, including cost-effectiveness of dupilumab compared to conventional treatment, patient’s preference following comprehensive counseling, the national healthcare infrastructure and geographical location [11, 17]. These considerations are crucial for ensuring that therapy is both patient-centered and resource-appropriate.

This study has a few limitations that needs attention. First, the study’s small sample size and single-center design may restrict the applicability of the findings to a broader population. Second, as a retrospective and observational study, there is an inherent risk of selection bias and unmeasured confounding factors. Third, we were able to evaluate only two of the five recommended EPOS/EUFOREA response criteria. The remaining criteria—improved olfactory function in objective smell testing, reduced impact of comorbid conditions, and no need of sCOS/salvage ESS—were unfortunately not routinely measured in our clinical setting [5]. Additionally, reliance on SNOT-22 subitem “smelling” to evaluate smell impairment, rather than standardized objective smell tests, as indication criterion for biologic treatment, represents another limitation. Lastly, the six-month follow-up period may be insufficient to assess the long-term effects of dupilumab treatment. Many patients continued their care in outpatient settings beyond the study period, limiting our ability to collect extended follow-up data. Despite these limitations, this study provides important real-world insights into the use of dupilumab for the treatment of CRSwNP.

## Conclusions

We found that dupilumab was effective in CRSwNP treatment, regardless of baseline nasal polyp size. Both small and large polyp groups showed significant improvements in NPS and patient-reported symptom scores. Future, prospective studies are warranted to validate these findings.

## Data Availability

The datasets generated and analyzed during the current study are available from the corresponding author on reasonable request.
